# Internet-Based Cognitive Behavior Therapy vs. Cognitive Behavioral Group Therapy for Social Anxiety Disorder: A Randomized Controlled Non-inferiority Trial

**DOI:** 10.1371/journal.pone.0018001

**Published:** 2011-03-25

**Authors:** Erik Hedman, Gerhard Andersson, Brjánn Ljótsson, Erik Andersson, Christian Rück, Ewa Mörtberg, Nils Lindefors

**Affiliations:** 1 Department of Clinical Neuroscience, Division of Psychiatry, Karolinska Institutet, Stockholm, Sweden; 2 Department of Behavioural Sciences and Learning, Swedish Institute for Disability Research, Linköping University, Linköping, Sweden; 3 Department of Psychology, Stockholm University, Stockholm, Sweden; University of Granada, Spain

## Abstract

**Background and Aims:**

Cognitive behavioral group therapy (CBGT) is an effective, well-established, but not widely available treatment for social anxiety disorder (SAD). Internet-based cognitive behavior therapy (ICBT) has the potential to increase availability and facilitate dissemination of therapeutic services for SAD. However, ICBT for SAD has not been directly compared with in-person treatments such as CBGT and few studies investigating ICBT have been conducted in clinical settings. Our aim was to investigate if ICBT is at least as effective as CBGT for SAD when treatments are delivered in a psychiatric setting.

**Methods:**

We conducted a randomized controlled non-inferiority trial with allocation to ICBT (n = 64) or CBGT (n = 62) with blinded assessment immediately following treatment and six months post-treatment. Participants were 126 individuals with SAD who received CBGT or ICBT for a duration of 15 weeks. The Liebowitz Social Anxiety Scale (LSAS) was the main outcome measure. The following non-inferiority margin was set: following treatment, the lower bound of the 95 % confidence interval (CI) of the mean difference between groups should be less than 10 LSAS-points.

**Results:**

Both groups made large improvements. At follow-up, 41 (64%) participants in the ICBT group were classified as responders (95% CI, 52%–76%). In the CBGT group, 28 participants (45%) responded to the treatment (95% CI, 33%–58%). At post-treatment and follow-up respectively, the 95 % CI of the LSAS mean difference was 0.68–17.66 (Cohen’s *d* between group = 0.41) and −2.51–15.69 (Cohen’s *d* between group = 0.36) favoring ICBT, which was well within the non-inferiority margin. Mixed effects models analyses showed no significant interaction effect for LSAS, indicating similar improvement across treatments (*F* = 1.58; df = 2, 219; *p* = .21).

**Conclusions:**

ICBT delivered in a psychiatric setting can be as effective as CBGT in the treatment of SAD and could be used to increase availability to CBT.

**Trial Registration:**

ClinicalTrials.gov NCT00564967

## Introduction

Social anxiety disorder (SAD), or social phobia, is characterized by a persistent and debilitating fear of being evaluated by others. SAD typically has an early onset [1], runs a chronic course [2], is one of the most prevalent anxiety disorder in the western world [3], and is associated with functional impairment [4]. Cognitive behavioral group therapy (CBGT) for SAD has proven to be effective in several randomized controlled trials (RCTs) over the last 20 years. Results have shown that CBGT is superior to psychological [5] and pill placebo and that it can be as effective as pharmacological treatment with SSRIs [6], making it the most established psychological treatment for SAD [7], [8]. However, CBGT is available to only a few due to a lack of trained therapists [9]. While individually administered CBT has demonstrated large effects [10] and might be more efficacious than CBGT [11], [12], this treatment format is even more dependent on the availability of trained therapists.

More recently, guided Internet-based CBT (ICBT) has shown promising results in RCTs conducted by three independent research groups [13], [14], [15], [16], [17], [18]. The treatment entails the same components as conventional CBT, such as exposure to feared situations, but is delivered over the Internet with therapist contact via an online messaging system resembling e-mail [14]. Evidence has shown that improvements made during ICBT are persistent [19]. ICBT has some important advantages over live treatment. First, it is not restricted in time or to a specific geographic location (i.e., patients as well as therapists can work with the treatment at any time or place they wish). Second, since ICBT demands less therapist time, ICBT therapists can treat significantly more patients than possible with CBGT [20], [21]. Consequently, ICBT has the potential to dramatically increase availability of CBT.

Although ICBT for SAD has demonstrated effects in line with CBGT, the current evidence holds a number of limitations. There has been no comparison to conventional CBT, such as CBGT, and most studies have relied solely on self-report instruments as measures of treatment outcome. In addition, most studies have been conducted in university settings, which might have a different impact on treatment experience and outcome compared to receiving care at a psychiatric clinic. Although one study has indicated that the characteristics of Internet clinic patients could be similar to those of outpatient clinics [22], the research field would benefit from a trial conducted in a psychiatric context. Finally, diagnostic procedures may be more clinically valid when conducted in a clinical setting. This has not been the case in the previous studies where only telephone interviews or self-report have been used.

In summary, more empirical evidence is needed before ICBT can be validly employed in a psychiatric context. As CBGT is an effective gold standard treatment appropriate for use as a benchmark [23], the necessary evidence to validate ICBT is to demonstrate non-inferiority (i.e., equal efficacy) to CBGT [24]. The aim of the present study was to determine whether ICBT is as effective as CBGT for patients with SAD when administered in a psychiatric setting. We hypothesized that ICBT would be at least as effective as CBGT in reducing social anxiety. We also predicted that the two treatments would be equal on secondary outcome measures of depressive symptoms, general anxiety, quality of life, and global functioning.

## Methods

### Trial design

The protocol for this trial and supporting CONSORT checklist are available as supporting information; see Checklist S1 and Protocol S1. This was a non-inferiority trial within the context of a parallel group study with unrestricted randomisation in 1∶1 ratio conducted in Sweden. Outcome assessors were blind to treatment status.

### Recruitment and selection

Recruitment for the study took place between 2007 and 2009. Participants were recruited by referral from primary care physicians and psychiatrists, and by self-referral to the psychiatric clinic at the Karolinska University Hospital in Stockholm, Sweden. Information about the treatment and the study was published on the official web page of the clinic (www.internetpsykiatri.se). There were no advertisements in newspapers or other media. The study protocol was approved by the Regional Ethical Review Board in Stockholm (Karolinska Institutet) and written informed consent was obtained from all participants after a detailed description of the study had been given.

To be eligible for inclusion potential participants had to meet the following criteria: (a) fulfill the DSM-IV [25] criteria of social anxiety disorder as assessed using the Structured Clinical Interview for DSM-IV axis I disorders (SCID-I) [26], (b) agree to undergo no other psychological treatment for the duration of the study, (c) have no history of CBT for the last four years, (d) have constant dosage two months prior to treatment of any prescribed medication for anxiety or depression and agree to keep dosage constant throughout the study, (e) have a primary diagnosis of SAD as assessed by the interviewing psychiatrist (individuals with comorbid disorders according to the Mini International Neuropsychiatric Interview (MINI) [27] were not excluded), (f) not currently meet the diagnostic criteria for substance abuse (g) have no history of psychosis or bipolar disorder, (h) not score >20 on the Montgomery Åsberg Depression Rating Scale-self report (MADRS-S) [28], (j) if criteria for major depression were met, have a score of less than 4 of 6 on the suicide ideation item of MADRS-S, and (k) not meet criteria for any personality disorders within cluster A (e.g. paranoid personality disorder) or B (e.g. antisocial personality disorder), which could interfere with the therapeutic process in group therapy.

**Figure 1:**
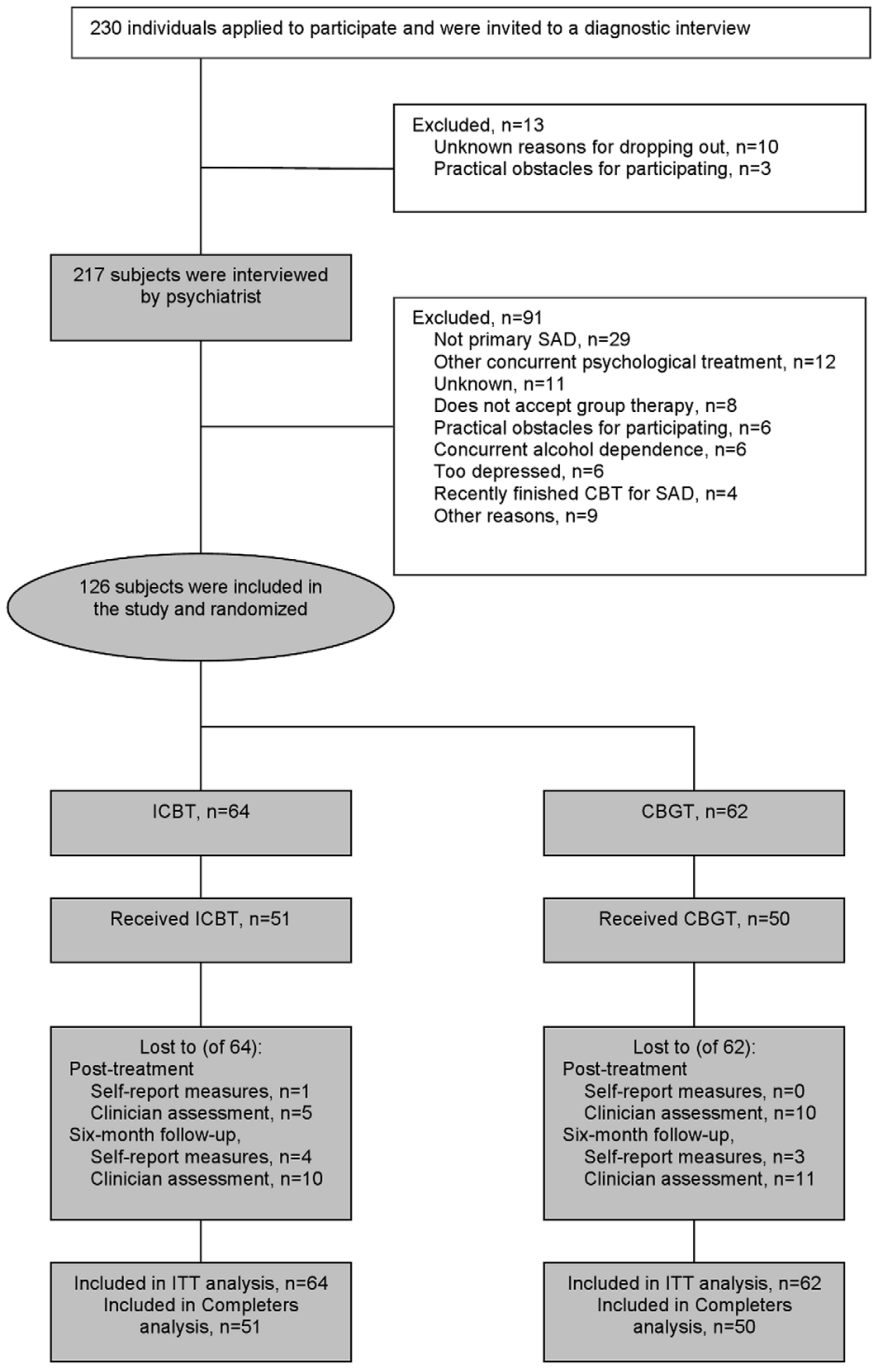
Participant flow and reasons for dropping out throughout the trial. Abbreviations: ICBT, Internet-based cognitive behavior therapy; CBGT, Cognitive behavioral group therapy; SAD, Social anxiety disorder; Received, completed at least 5 modules (ICBT) or 5 sessions (CBGT).

During the first stage of the recruitment process, potential participants were asked to complete the Social Phobia Screening Questionnaire (SPSQ) [29], MADRS-S, the Alcohol Use Disorders Identification Test (AUDIT) [30], and the Drug User Disorders Identification Test (DUDIT) [31]. In the second stage, participants were invited to attend an interview with a psychiatrist at the Karolinska University Hospital to confirm the SAD diagnosis and establish whether they met the remaining inclusion criteria (b-k). The psychiatrists conducting the assessments had more than 10 years of experience working with structured diagnostic interviews and had undergone extensive training in the use of the primary outcome measure, as well as of the SCID and the MINI. Two hundred thirty applicants completed the screening questionnaires and underwent the interview. Of those, 126 met all 10 inclusion criteria. Figure 1 shows the participant flow throughout the trial. Demographic information for participants is presented in Table 1.

**Table 1:** Demographic description of the participants.

Variable		ICBT	CBGT
		n = 64	n = 62
**Gender**	Women (%)	24.0 (37.5)	21.0 (33.8)
	Men (%)	40.0 (62.5)	41.0 (66.1)
**Age**	Mean age (SD)	35.2 (11.1)	35.5 (11.6)
	Min-max	20.1–63.2	18.0–64.1
**Social anxiety disorder**	Generalized subtype (%)	56.0 (87.5)	53.0 (85.5)
	Mean duration, years (median)	20 (18)	21.95 (21.5)
	Mean age of onset	15.6	13.1
**Occupational status**	Working 75–100 % (%)	51 (79.7)	42 (67.7)
	Sick leave, part or full time (%)	4 (6.3)	6 (9.7)
	Disability pension (%)	2 (3.2)	1 (1.6)
**Referral**	From out patient clinics	16 (25)	13 (21.0)
	Self-referral	48 (75)	49 (79)
**Stabilized psychotropic medication**	SSRIs	14	11
	SNRIs	2	4
Comorbid psychiatric disorders	Any anxiety disorder	10	12
	Major depression	10	9
	Avoidant personality disorder	33	29

Abbreviations: ICBT, Internet-based Cognitive Behavior Therapy; CBGT, Cognitive Behavior Group Therapy; SSRI, Selective serotonin reuptake inhibitor; SNRI, Serotonin–norepinephrine reuptake inhibitor.

### Outcome measures

#### Social anxiety

The primary outcome measure was the clinician administrated Liebowitz Social Anxiety Scale (LSAS) [32], [33]. The LSAS has good psychometric properties including high internal consistency (Cronbach’s *α* = .96) and high test-retest reliability over one week (*r* = .97) [32], [34]. The self-report version of LSAS (LSAS-SR) [35] was used as a complement during the treatment phase The LSAS-SR has a high internal consistency (Cronbach’s *α* = .95) as well as a high 12-week test-retest reliability (*r* = .83) [32], [35]. The Social Phobia Scale (SPS) [36] and the Social Interaction Anxiety Scale (SIAS) [36] were also administered to assess a broader spectrum of social anxiety symptoms. Both scales have good psychometric properties [36].

#### General anxiety, anxiety sensitivity, depression and quality of life

The Beck Anxiety Inventory (BAI) [37] and the Anxiety Sensitivity Index (ASI) [38] were used to assess general anxiety and anxiety sensitivity. The Montgomery Åsberg Depression Rating Scale-self report (MADRS-S) [28] was used to measure depressive symptoms. Finally, the Quality of Life Inventory (QOLI) [39] was used to assess quality of life. The BAI, ASI, MADRS-S and the QOLI have all demonstrated good psychometric properties.

#### Diagnostic assessment, global functioning and improvement

Psychiatric diagnoses were established using the SCID-I-Research version (RV) [26], the SCID-II [40], and the MINI [27]. The SCID-I-RV was used to assess SAD since it has the advantage of providing information in greater detail than the MINI (which was not used to assess criteria for SAD) and has high inter-rater reliability [41]. To assess avoidant personality disorder, we used SCID-II, which has very good inter-rater reliability [42]. The MINI was used to assess axis I disorders other than SAD [27]. Assessors used the Global Assessment of Functioning Scale (GAF) [25] to measure global functioning and the Clinical Global Impression Improvement Scale (CGI-I) [43] to measure global improvement.

#### Assessment of treatment credibility and treatment preference

A treatment credibility scale comprising five items was administered to determine whether participants viewed the two treatments as equally credible [44]. Prior to randomization participants were asked to state their treatment preference (ICBT or CBGT).

### Administration format of self-report measures

We used an Internet application to administer the LSAS-SR, SIAS, SPS, BAI, MADRS-S, and the QOLI. Internet and paper-and-pencil administrations of these measures have been shown to possess equivalent psychometric properties [45].

### Procedure

#### Assessment points and randomization

Assessments, including diagnostic interviews, were conducted before treatment (i.e., pre-treatment), immediately after treatment (i.e., post-treatment), and six months after treatment (i.e., follow-up). During treatment, the LSAS-SR and the suicide ideation item of MADRS-S were administered on a weekly basis. Participants assessed treatment credibility after one week of treatment. The randomization procedure involved two external persons not involved in the study; one provided randomization data and the other monitored that no manipulation of treatment allocation was performed by the research group. A true random number service (http://www.random.org) was used to ensure randomization. The random sequence was generated after inclusion of participants to ensure that assignment of intervention was concealed from assessing psychiatrists and researchers of the study. Participants were allocated to CBGT or ICBT in a 1∶1 ratio using simple randomization with no restrictions or matching. To ensure the integrity of the blinding procedure, participants were instructed not to mention which treatment they had received during the post-treatment and follow-up interviews. After completing the interviews, the assessing psychiatrists guessed allocation status for each participant.

#### Monitoring of treatment integrity

Treatment integrity of CBGT was ensured in three ways. First, we used a detailed treatment manual [46]. Second, group therapists received supervision throughout the trial by a licensed psychotherapist specialized in CBT for SAD. Third, all sessions were audio recorded and a random sample of 5 sessions was audited by a clinical psychologist with more than 10 years of experience in treating SAD with CBT. Using the Therapist Adherence Scale (TAS) developed by the originators of CBGT [47], all reviewed sessions were judged to have been conducted in accordance with the treatment manual. The average TAS score of the reviewed session was 4.5 (SD = 0.5) on a 1 (ineffective) to 5 (extremely effective) scale. Due to the fixed nature of ICBT and the limited role of the therapist, no measure of treatment integrity was taken for ICBT. However, all therapists who provided the guidance of ICBT received supervision from a clinical psychologist throughout the trial and all therapists had previous experience of that treatment format.

### Treatments

#### Internet-based cognitive behavior therapy (ICBT)

The ICBT employed in this study was based on the treatment developed by Andersson and coworkers, and has been validated by several randomized controlled trials [13], [14], [15]. The treatment followed a CBT-model, developed for individual therapy, that stresses the importance of avoidance and safety behaviors as well as misinterpretations of social events and internal focus as maintaining factors of SAD [48]. A vital part of the treatment was the gradual access to internet-based self-help text comprising 15 text modules, each covering a specific theme (e.g., exposure or cognitive restructuring) completed with a homework component. The modules provided the participants with the same knowledge and tools as conventional individual CBT for SAD.

The duration of ICBT was 15 weeks and throughout this period the patient had access to a therapist via an online secure messaging system. The role of the therapist was mainly to provide feedback regarding home work and to grant access to the treatment modules. However, the patient could contact the therapist at any time and expect a reply within 24 hours during week days. Patients and therapists had no face-to-face or telephone contact during the treatment. The general instruction to the internet therapists was to have the ambition to restrict time spent on each patient to less than 10 minutes per week. This time frame was judged possible as most messages to patients are very brief entailing the core feed-back that the homework was successfully completed and the next treatment module is accessible. The therapists conducting ICBT were eight psychologists with one to four years of experience in delivering CBT via the internet.

#### Cognitive behavioral group therapy (CBGT)

This treatment comprised an initial individual session followed by 14 group sessions over 15 weeks. The individual session prepared the participant to begin group treatment sessions and included a rationale for group treatment. Each group session was 2.5 hours long, including a 15 minute break. Groups were lead by two therapists and had six to seven participants. The CBGT followed the protocol developed by Heimberg and Becker [46] with the addition of two group sessions. The first two group sessions (i.e., Sessions 2–3) were aimed at teaching participants the role and components of anxiety and how to identify and challenge negative automatic thoughts. Sessions 4–14 focused primarily on individually tailored in-session exposure in combination with cognitive restructuring. Prior to exposure exercises, participants identified and disputed negative automatic thoughts, developed rational alternatives, and behavioral goals were set. Following the exposure exercises, additional cognitive restructuring was conducted and goal attainment was reviewed. Participants were also given homework to continue exposure exercises in the same fashion in their home environment. Session 14–15 were devoted to assessing the progress of the participant and setting goals for the future. A detailed plan was created for each participant to ensure that goals and methods to achieve them were clear. The therapists facilitating the CBGT sessions were six clinical psychologists with 2 to15 years experience in treating patients with SAD using CBT.

### Statistical analysis

Statistical analyses were conducted using PASW version 18.0 (SPSS inc., Chicago). The non-inferiority margin of the primary outcome measure LSAS was set at Δ10 points, which was based on clinical judgment and a review of the evidence of CBGT compared to credible control conditions for SAD. Meta-analytic reviews, adopting random-effects models, have estimated the lower bound of the 95% confidence interval (CI) of the between group effect size to 0.39 (Hedges’*g*) [49]. Assuming a standard variance of LSAS scores in our sample, this supported the use of 10 LSAS points as a non-inferiority margin. Test criterion for non-inferiority was that the lower bound of the 95% CI of the mean difference should fall within Δ. With 95% probability, the mean difference between ICBT and CGBT had to be smaller than 10 LSAS points. As this was a non-inferiority trial, this criterion did not apply for the upper bound of the CI, meaning that the CI could exceed 10 LSAS points if in favor of ICBT. For the other continuous measures, the non-inferiority margin was set at Δ Cohen’s *d* = 0.5. Test criterion for non-inferiority for these measures was that the lower bound of the 95% CI of between group effect sizes should fall within this range. This criterion was judged acceptable as it has been proposed that an effect size of 0.5 marks the border between a mild and moderate effect [50]. Thus, this criterion meant that mild effects up to the border of moderate effects were acceptable.

Main outcome continuous variables were analyzed using a linear mixed effects model because of its superior qualities regarding missing data as well as in reducing the risk of committing type I errors [51]. We employed the restricted maximum likelihood method assuming a compound symmetry model as covariance structure since it provided the best model in an information criteria comparison. T-tests were used to compare treatment credibility ratings and χ^2^ tests to assess nominal scale variables. Wilcoxon tests and Mann-Whitney U tests were used to analyze outcomes on ordinal scale variables. To estimate rates of responders, we used the clinical significant improvement criteria as suggested by Jacobson and Truax on the primary outcome measure [52]. The criteria for clinical significant improvement was to have a score below 43.3 (closer to healthy population than to SAD population [53] and the reliable change criterion was established using the test-retest reliability coefficient of .97 [34]. Cohen’s *d* based on pooled standard deviations was used to calculate effect sizes. The sample size was considered satisfactory since power calculations showed that there was a chance slightly lower than 80% to detect a difference, given the non-inferiority criteria used and an alpha-level of .05. The main analyses were conducted in accordance to intention-to-treat principle, i.e. all available assessment data was analyzed in accordance to how participants were randomized. This meant that participants were encouraged to provide assessment data regardless of treatment adherence. On the CGI-I scale, missing values were replaced with “no change”. As a complement, the social anxiety measures were also analyzed based on the sample of completers only. There were no significant differences between the groups on the outcome measures at baseline (*t*_(1, 124)_ = 0.02–1.38, p = .17–.98).

## Results

### Attrition

Loss of data is presented in Figure 1.

### Effect sizes and non-inferiority

Within group effect sizes on the primary outcome measure LSAS were large for both treatments. At post-treatment and six month follow-up respectively, the 95 % CI of the mean difference between the groups on LSAS score was 0.68–17.66 and −2.5–15.69, favoring ICBT. This was well within the non-inferiority margin of 10 LSAS points for the lower bound. Analysis of the other continuous measures showed that all lower bounds of 95% CIs for between group effect sizes fell well within the non-inferiority margin of *d* = 0.5 effect sizes. As stated above, Table 2 provides within and between group effect sizes on continuous outcome measures.

**Table 2:** Means, SDs and effect sizes (Cohen’s *d*) for measures of social anxiety and secondary outcome variables.

					Effect size	Effect size	Effect size	Effect size
Measure	Group	Pre	Post	FU	Between	Between	Whithin	Within
		M (SD)	M (SD)	M (SD)	Post (95% CI)	Follow-up (95% CI)	Pre-Post	Pre-FU
**LSAS**	ICBT	68.4 (21.0)	39.4 (19.9)	32.1 (23.1)			1.42	1.64
					0.41 (0.03–0.78)	0.36 (−0.02–0.75)		
	CBGT	71.9 (22.9)	48.5 (25.0)	40.7 (23.7)			0.97	1.34
**SIAS**	ICBT	46.2 (16.8)	34.6 (15.1)	29.9 (15.7)			0.73	1.01
					0.24 (−0.11–0.59)	0.33 (−0.03–0.69)		
	CBGT	49.3 (14.8)	38.53 (15.7)	34.6 (15.1)			0.72	0.93
**SPS**	ICBT	32.8 (14.6)	21.6 (13.5)	17.6 (13.9)			0.80	1.06
					0.04 (−0.31–0.39)	0.16 (−0.20–0.51)		
	CBGT	33.5 (14.0)	22.1 (14.3)	19.7 (13.6)			0.80	0.99
**MADRS-S**	ICBT	12.7 (6.5)	9.1 (6.9)	8.8 (8.3)			0.53	0.52
					0.29 (−0.06–0.64)	0.21 (−0.15–0.57)		
	CBGT	14.0 (8.0)	11.5 (8.8)	10.5 (8.6)			0.30	0.41
**BAI**	ICBT	18.7 (10.9)	12.1 (8.6)	10.6 (10.0)			0.67	0.77
					0.21 (−0.14–0.56)	0.13 (−23–0.48)		
	CBGT	18.6 (10.8)	14.2 (11.3)	11.8 (9.2)			0.40	0.77
**QOLI**	ICBT	0.8 (1.5)	1.6 (1.6)	1.8 (1.5)			0.51	0.69
					0.27 (−0.08–0.62)	0.46 (0.10–0.82)		
	CBGT	0.4 (1.6)	1.1 (1.7)	1.1 (1.5)			0.45	0.47
**ASI**	ICBT	22.6 (11.0)	16.1 (10.8)	14.4 (11.3)			0.59	0.73
					0.14 (−0.21–0.49)	−0.11 (−0.44–0.27)		
	CBGT	22.0 (10.0)	17.6 (10.7)	13.6 (8.7)			0.42	0.89

Abbreviations: ICBT, Internet-based Cognitive Behavior Therapy; CBGT, Cognitive Behavior Group Therapy; Pre, before treatment; Post, post-treatment; FU, six months after treatment; LSAS, Liebowitz Social Anxiety Scale; SIAS, Social Interaction Scale; SPS, Social Phobia Scale; MADRS-S, Montgomery-Åsberg Depression Rating Scale-Self report; BAI, Beck Anxiety Inventory; QOLI, Quality of Life Inventory; ASI, Anxiety Sensitivity Index.

### Treatment effectiveness - primary outcome measure (LSAS)

**Figure 2:**
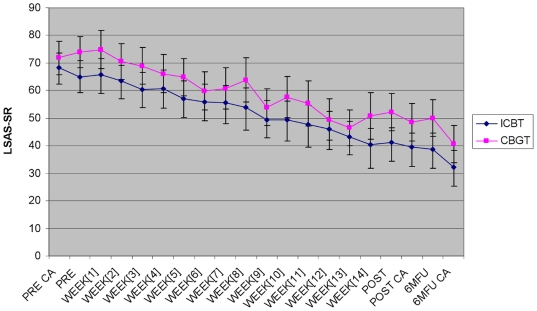
Weekly change on LSAS-SR during treatment and LSAS scores at each assessment point. Abbreviations: ICBT, Internet-based cognitive behavior therapy; CBGT, Cognitive behavioral group therapy; LSAS, Liebowitz Social Anxiety Scale; -SR, Self-report; CA, Clinician administered; Pre, Before treatment; Post, Post-treatment; 6MFU, Six months after treatment.

At post-treatment, 35 (55%) of the participants (95% CI, 42.5%–66.9%) in the ICBT group were classified as responders according to the Jacobson and Truax criteria [52] compared to 21 participants (34%) in the CBGT group (95% CI, 22.1%–45.7%). At six-month follow-up, the corresponding number was 41 (64%) in the ICBT group (95% CI, 52.3%–75.8%) and 28 (45%) in the CBGT group (95% CI, 32.8%–57.6%). Mixed effects model analysis showed a significant effect of time, indicating improvement in both treatment groups (*F* = 179.06; df = 1,219; *p*<.001). There was no significant interaction of group and time for the primary outcome measure LSAS, indicating similar improvement across groups (*F* = 1.58; df = 2, 219; *p* = .21). As illustrated in Figure 2, there were continuous within group improvements throughout the trial on the LSAS-SR in both conditions. The means (SDs) on the LSAS-SR at pre, post and follow-up respectively were 65.0 (23.6), 41.1 (21.5), 38.7 (23.1) for the ICBT group and 73.9 (21.5), 52.2 (25.5), 50.0 (24.9) for the CBGT group. There was no significant interaction of group and time for the LSAS-SR (*F* = 0.25; df = 2, 243; *p* = .77). Table 2 provides within and between group effect sizes on measures of social anxiety, depression, general anxiety, quality of life and anxiety sensitivity.

### Treatment effectiveness - secondary outcome measures

#### Social anxiety

There was a significant effect of time on both the SIAS and SPS (*F* = 80.95 −83.39; df = 2, *p*<.001). Mixed effects model analysis showed no significant interaction of group and time for these variables (*F* = 0.30–0.48; df = 2, 244; *p* = .62–.74).

#### Depression, general anxiety, anxiety sensitivity and quality of life

There was a significant effect of time on MADRS-S, BAI, ASI and QOLI (*F* = 17.26–52.30; df = 2, 227–245; *p*<.001). Analysis using mixed effects model yielded no significant interaction of group and time for these variables (*F* = 0.26–1.30; df = 2, 227–245; p = .28–.77).

#### Clinician administrated measures of global improvement and functioning

**Figure 3:**
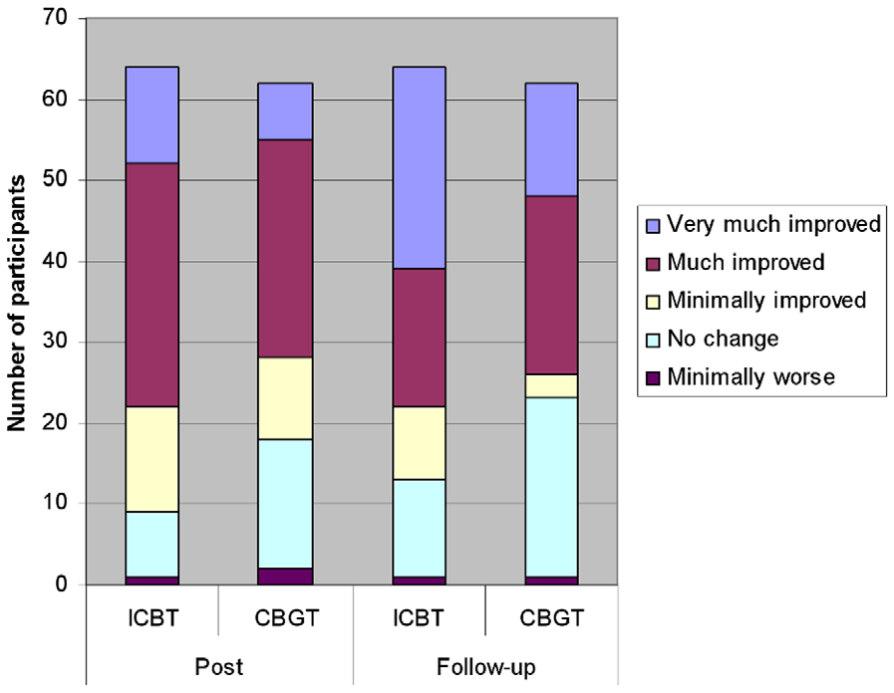
CGI-I scores at post-treatment and six-month follow-up. Abbreviations: ICBT, Internet-based cognitive behavior therapy; CBGT, Cognitive behavioral group therapy; CGI-I, Clinical Global Impression Improvement Scale.

At post-treatment, 42 participants (66%) in the ICBT group were classified as very much improved or much improved according to the CGI-I (95% CI, 59.1%–81.5%). In the CBGT group, the corresponding number of participants was 34 (55%) as assessed using the CGI-I (95% CI, 42.5%–67.2%). Wilcoxon tests showed that participants who had received ICBT were further improved at follow-up according to the CGI-I (Z = 2.33, *p*<.02), but CBGT participants were not (Z = 1.50, p = .14). Mann-Whitney U-test showed no significant difference between ICBT and CGBT at post-treatment (*p* = .08.). However, at six-month follow-up, participants receiving ICBT were significantly more improved on the CGI-I (p<.01). Figure 3 displays CGI-I scores at post-treatment and follow-up. The means (SDs) on the GAF at pre, post and follow-up respectively were 57.3 (9.8), 66.8 (10.0), 70.4 (11.3) for the CBGT group and 59.5 (6.4), 69.7 (10.8), 74.5 (11.6) for the ICBT group. Using a mixed effects model approach, no significant interaction of time and treatment group was found on the GAF (*F* = 0.354; df = 2, 225; *p* = .59).

#### Psychiatric diagnosis at each assessment point

Following treatment, 18 (31 %) participants who had received ICBT no longer met diagnostic criteria for SAD (28 % if considering dropouts as non-responders). The corresponding number for participants who underwent CBGT was 12 (23 %; 19 % if considering dropouts non-responders). At follow-up, 25 (46%) participants who had received ICBT (41% if considering dropouts non-responders) and 21 (40%) receiving CBGT (34% if considering dropouts non-responders) no longer met diagnostic criteria for SAD. At post-treatment and six month follow-up there was no significant difference in the prevalence of SAD between groups (χ^2^ = 037–1.33, df = 1, *p* = .25–.55).

### Treatment credibility

Credibility ratings after one week of treatment showed that participants in both groups rated their respective treatment as credible. Out of a possible total of 50, the average scores were 34.0 (SD = 9.03) and 33.8 (SD = 10.6) for ICBT and CGBT, respectively. There was no significant difference in treatment credibility between treatment groups (*t*_(1, 110)_ = 0.07, p = .95).

### Assessment of blinding and treatment preference

In four instances the blinding was broken. On two occasions participants accidentally mentioned their treatment allocation status to the assessor, and in other two occasions it was deemed necessary to break the blinding because of the need to assess increased depressive symptoms during treatment. As shown in Table 3, there was no significant association between assessors’ guess and actual treatment allocation (χ^2^ = 0.27, df = 1, *p* = .61), indicating successful blinding.

**Table 3:** Agreement between actual treatment status and assessors’ guess (expected frequency).

Assignment	Assessors' guess		
	ICBT	CBGT	Total
ICBT	38 (36.6)	26 (27.4)	64
CBGT	34 (35.4)	28 (26.6)	62
Total	72	54	126

Abbreviations: ICBT, Internet-based Cognitive Behavior Therapy; CBGT, Cognitive Behavior Group Therapy.

Prior to randomization participants were asked to state their treatment preference. Of 126 participants, 68 (54%) preferred ICBT and 58 (46%) CBGT. There was no difference between groups in terms of proportion of participants that received the preferred treatment (χ^2^ = 0.77, df = 1, *p* = .38).

### Treatment adherence

In CBGT, the average number of attended sessions per participant was 9.40 (SD = 4.87) out of a possible total of 15. Fifty participants in CBGT (81%) attended at least five sessions and 17 (27%) attended all sessions. The average number of completed modules in ICBT was 9.33 (SD = 4.95) of 15. Fifty-one participants in ICBT (80%) completed at least 5 modules and 19 (29.7%) completed all modules. Important to note is that the main components of the treatments had been introduced at week 5.

### Evaluation of therapist resources required for each treatment

On average, therapists delivering ICBT spent 5.5 minutes (SD = 3.6) weekly per patient. The corresponding amount of time in CBGT was 50 minutes (2.5 h sessions with two therapists and 6 patients). Taking nonattendance into consideration, this number would have been even higher. ICBT therapists sent 17.4 messages to each patient on average, i.e. 1.2 weekly per patient.

### Intention-to-treat vs. treatment exposed analysis

Analysis including only those exposed to treatment (at least five sessions or modules) yielded results equal to the intention-to-treat analysis on continuous outcome measures of social anxiety, indicating that between group effects in the latter were not moderated by completion status. There was a significant effect of time on all three measures (*F* = 90.52 -188.67; df = 2, 188–198; *p*<.001). Mixed effects models analysis showed no significant interaction effect of time and treatment group on the LSAS (*F* = 1.78; df = 2, 188; *p* = .17), nor on the SIAS and the SPS (*F* = 0.32–0.43; df = 2, 198; *p* = .66–.73).

## Discussion

The present study is the first to demonstrate that ICBT can be as effective as CBGT in the treatment of SAD. Both treatments demonstrated large within group effect sizes on measures of social anxiety and general anxiety. The confidence interval of mean differences of the primary outcome measure fell well within the non-inferiority margin and between-group effect sizes were small but consistently favoring ICBT on the social anxiety measures. There was also a large proportion of participants who were classified as much improved or very much improved at post-treatment and follow-up in both treatment groups. The indication that the ICBT group was slightly more improved on the CGI-I should be interpreted cautiously as effects were small and no alpha-level corrections were set. The follow-up assessment indicated that treatment gains were sustained on all measures. These results indicate that ICBT can be an effective treatment for patients with SAD when delivered in a regular psychiatric setting.

In trials assessing non-inferiority it is essential that the effect of the gold standard treatment is as effective as in previous trials. This was the case in the present study, where CBGT yielded effects in line with trials conducted by its originators [54]. Moreover, treatment effects for ICBT were equivalent to those reported in previous controlled trials [13], [14], [15], [17], [18]. These are strengths of the present study. As reduced therapist time is an important element of ICBT, a key finding in this study is that ICBT reduced therapist time per treated patient by 90% compared to CBGT. As previously stated, individual CBT developed by Clark and Wells (1995) may be even more effective than CBGT. It could therefore be argued that Clark’s individual cognitive therapy should be the benchmark against which ICBT is contrasted. However, as CBGT has been evaluated in more trials and is more established, we decided to use CBGT as the benchmark treatment.

Overall, we interpret the results of the present study as indicating that a substantial proportion of persons with SAD respond well to ICBT. However, for those who do not respond to ICBT, an intensified treatment such as individual face-to-face CBT might be superior. Thus, we view ICBT as a complement (not a substitute) to conventional CBT that could facilitate the dissemination of CBT and improve healthcare resource allocation. When results are interpreted it is also important to bear in mind that that the non-inferiority margin allowed for up to a moderate effect between treatments. However, this margin was judged as clinically valid, and again, if ICBT is to be used as a complement, the usefulness of employing very narrow non-inferiority margins is limited.

There are several limitations that warrant mention and could provide venues for future research. First, there was no randomization to an active placebo condition, which raises the issue of misinterpreting regression to the mean as indicative of two effective treatments. However, given the chronicity demonstrated by SAD [55], high proportions of spontaneous remission is improbable. In addition, meta-analytic evidence suggests CBT for anxiety disorders tends to be more effective than placebo [49]. A second limitation concerns patient preferences. It is likely that participants in our trial were willing to receive either ICBT or CBGT, which may not be representative for persons with SAD in the general population. It may be that internet treatment is preferred over group treatment. Despite the observation that persons with SAD are frequent internet users [56], this has not yet been studied. Third, the current study did not include long-term follow-up and, as such, it cannot be determined if the effects of the CBGT and ICBTs protocol for SAD are enduring over durations longer than six months. However, previous studies of CBGT [57] and ICBT suggest that the results may be enduring [19]. The adherence rate also deserves mentioning. Eighty percent of the participants completed at least the first five weeks of therapy and were thereby exposed to the main components of the treatment, although significantly fewer completed all sessions or modules. Still, we find that having completed five weeks is an important threshold as preliminary analyses of outcome predictors have shown that completing at least five weeks is associated with better outcome, whereas completing all sessions or modules seems to yield little additional effect. As for CBGT, this adherence rate is comparable to that of a recently conducted large scale RCT where 35% of CBGT participants discontinued treatment [58]. Finally, we did not assess treatment satisfaction in the present study. However, data from regular care of the Internet clinic where this study was conducted suggest high satisfaction with treatment as the average score on the Client Satisfaction Questionnaire [59] is 3.18 (SD = 0.57) using a scale range of 1–4.

In spite of these limitations, we conclude that ICBT may be at least as effective as CBGT, and that it is feasible to conduct ICBT for SAD in a psychiatric setting. As ICBT requires much less resources than conventional CBT, it could be the most promising means to increase the availability of CBT for persons affected by SAD.

## Supporting Information

Protocol S1 **Trial Protocol.**
(DOC)

Checklist S1 **CONSORT Checklist.**
(DOC)
