# Supplementary material for: Internet-Based Cognitive Behavior Therapy vs. Cognitive Behavioral Group Therapy for Social Anxiety Disorder: A Randomized Controlled Non-inferiority Trial
Source: PLoS One. 2011 Mar 25;6(3):e18001. doi: 10.1371/journal.pone.0018001 (PMC3070741; doi:10.1371/journal.pone.0018001)
Supplement: Protocol S1 — Trial Protocol. (DOC) [file pone.0018001.s001.doc]

# ANSÖKAN OM ETIKPRÖVNING

# se anvisning sid. 16

____________________________________________________

## **Uppgifter som fylls i av den regionala etikprövningsnämnden**

Ankomstdatum:       Dnr: PROTOCOL S1

Avgift inbetald datum:       Begäran om komplettering

av ansökan:

Ansökan komplett:

Begäran om ytterligare

Begärd information inkommen:       information:

Expeditionsdatum:       Beslutsdatum:

________________________________________________________________________

## **Uppgifter som fylls i av sökanden**

Till Regionala etikprövningsnämnden i: Stockholm

(Den regionala etikprövningsnämnd till vars upptagningsområde forskningshuvudmannen hör,

se www.forskningsetikprovning.se)

Ange en beskrivande titel på svenska för lekmän, utan sekretesskyddad information.

Ange också i förekommande fall projektets identitet, projektets/forskningsplanens (protokollets eller prövningsplanens) nummer, version, datum osv.

### Projekt

Jämförelse mellan Internetbaserad självhjälpsbehandling och gruppbehandling vid social fobi - En klinisk prövning med kognitiv beteendeterapi (KBT).

Projektnummer/identitet:       Version nummer:

**Ansökan avser (gäller även vid begäran om rådgivande yttrande):**

forskning där endast en forskningshuvudman deltar (5000 kr)

forskning där fler än en huvudman deltar (16000 kr)

forskning där mer än en forskningshuvudman deltar, men där samtliga

forskningspersoner eller forskningsobjekt enligt 4 § lagen (2003:460) om

etikprövning av forskning som avser människor, har ett omedelbart

samband endast med en av forskningshuvudmännen (5000 kr)

endast behandling av personuppgifter (5000 kr)

forskning som gäller klinisk läkemedelsprövning (16000 kr)

ändring av tidigare godkänd ansökan (enligt 4 §) (2000 kr)

Om nämnden finner att studien/forskningsprojektet inte faller inom lagens för etikprövning tillämpningsområde önskas ett rådgivande yttrande

Ja:  Nej:

**1. Information om forskningshuvudman m.m.**

1:1 Sökande forskningshuvudman

Ansökan om etikprövning av forskning skall göras av forskningshuvudmannen. *Med forskningshuvudman avses en statlig myndighet eller en fysisk eller juridisk person i vars verksamhet forskningen utförs.* Inom staten utförs forskning främst vid lärosätena, men även vid vissa andra myndigheter, som t.ex. Brottsförebyggande rådet och Socialstyrelsen. Kommuner och landsting kan vara forskningshuvudmän, liksom privaträttsliga juridiska personer.

Namn: Psykiatri Centrum Karolinska

Adress: Karolinska sjukhuset, 171 76 Stockholm

**1:2 Behörig företrädare**

.

Behörig företrädare för forskningshuvudmannen (t.ex. prefekt, enhetschef, verksamhetschef). Forskningshuvudmännen bestämmer själva, genom interna arbets- och delegationsordningar eller genom fullmakt, vem som är behörig att företräda forskningshuvudmannen. Bifoga kopia av sådan handling.

Namn: Peter Nordström Tjänstetitel: Chefsöverläkare

Adress: Psykiatri Centrum Karolinska, Karolinska sjukhuset, 171 76 Stockholm

**1:3 Forskare som är huvudansvarig för genomförandet av projektet (kontaktperson)**

Namn: Nils Lindefors, docent, bitr chefsöverläkare

Adress: Psykiatri Centrum Karolinska, Karolinska sjukhuset, 171 76 Stockholm

E-postadress: nils.lindefors@karolinska.se

Telefon: 08-51775013

Mobiltelefon: 0708-326208

**1:4 Plats**

Plats/er där projektet skall genomföras (ange inrättning/ar, institution/er, klinik/er etc.).

Psykiatri Centrum Karolinska

**1:5 Andra medverkande**

Övriga deltagande forskningshuvudmän samt forskare ansvariga att lokalt genomföra projektet (kontaktpersoner) skall anges i bilaga (namn, adresser).

Ej applicerbart

**1:6 Vid läkemedelsprövning**

Ansökan om tillstånd har insänts till Läkemedelsverket.

Ansökan inlämnad (datum):       Tillstånd erhållits

**1:7 Vid viss genetisk forskning**

Anmälan till *Datainspektionen* om förhandskontroll av behandling av personuppgifter om genetiska anlag som framkommit efter genetisk undersökning (10 § första stycket 2 personuppgiftsförordningen (1998:1191).

Inlämnad (datum):       Kommer att inlämnas efter godkänd etikprövning

### 2. Uppgifter om projektet

**2:1 Sammanfattande beskrivning av forskningsprojektet (programmet)**

Beskrivningen skall kunna förstås av nämndens lekmän. Undvik därför terminologi som kräver specialkunskaper.

Ange bakgrund och syfte för studien samt den (de) vetenskapliga frågeställning (ar) som man söker svar på. Ange de viktigaste undersökningsvariablerna. Ange vilka kunskapsvinster projektet kan förväntas ge och betydelsen av dessa. Ange om det är en registerstudie, uppdragsforskning etc.

För fackmän avsedd detaljerad information i protokoll eller forskningsplan *skall* bifogas som bilaga. En utförligare beskrivning över genomförandet *avsedd för lekmän* kan vid behov bifogas den för fackmän avsedda obligatoriska forskningsplanen.

Social fobi är en av de vanligaste ångeststörningarna med en livstidsförekomst på 7-13% i västvärlden. Prevalenssiffrorna varierar dock avsevärt mellan olika studier, sannolikt beroende på att man använt sig av olika diagnoskriterier, urvalsgrupper och analysmetoder, och det saknas också konsensus kring när den sociala rädslan bör definieras som patologisk. Strikta kriterier för betydande funktionsnedsättning och vad som är att betrakta som ett behandlingskrävande tillstånd resulterar i lägre prevalenstal. Även med en, sannolikt underskattad, punktprevalens (antal personer som uppfyller diagnoskriterier vid en given tidpunkt) på 1% innebär det dock att drygt 17.000 människor i Stockholms län idag kan antas lida av en funktionsnedsättande social fobi.

Debuten sker vanligtvis i tonåren och samsjukligheten med andra psykiatriska tillstånd, huvudsakligen ångest- och förstämningssyndrom samt alkoholrelaterade problem, är hög. Utan behandling är syndromet ofta kroniskt och konsekvenserna av den sociala fobin medför stora problem, både beträffande individuellt lidande men också ur ett samhällsekonomiskt perspektiv.

Kognitiv beteendeterapi (KBT) är den icke-farmakologiska behandlingsform som varit föremål för flest studier i sammanhanget och det finns idag klar evidens för att behandlingen har en god effekt. Behandlingen uppskattas medföra en meningsfull förbättring hos ca tre av fyra patienter och likvärdiga effektstorlekar har uppvisats för individuell behandling och behandling i gruppformat. Då tillgången på KBT-behandlare i Sverige är begränsad är det få patienter som erhåller effektiv psykologisk behandling. Det är därför angeläget att utvärdera alternativa behandlingsformer som kan reducera terapeutinsatser och behandlingstid, för att frigöra värdefull behandlartid så att fler patienter kan erbjudas effektiv vård.

Ett sätt att effektivisera vården, som i begränsad omfattning redan erbjuds inom psykiatrin, är att erbjuda behandling i gruppformat. Ytterligare ett alternativ är att överföra traditionella KBT-interventioner till Internetadministrerad självhjälpsbehandling med terapeutstöd via e-post (här kallad Internetbehandling). Metoden har prövats experimentellt vid Institutionen för psykologi, Uppsala Universitet, och goda resultat har uppvisats vid behandling av flera olika psykiska störningar.

Psykiatri Centrum Karolinska har under det senaste året genomfört ett pilotprojekt med Internetbehandling för paniksyndrom (KI-FoEtikKom-Nord D.nr 03-042). Resultaten var mycket goda med 17 av 20 patienter (85%) som efter avslutad behandling var fria från panikattacker. Rekryteringen har nu påbörjats till en randomiserad studie (D.nr. 04-034/4) där man jämför Internetbehandlingen med traditionell kognitiv beteendeterapi i gruppformat.

Preliminära resultat finns också från Uppsala Universitet där man genomfört två ännu opublicerade kontrollerade studier av Internetbehandling för social fobi (Etikprövningsnämnden i Uppsala, Dnr: 02-555 och 03-636). Studierna, som jämförde Internetbehandlingen mot obehandlad kontrollgrupp på väntelista, gav statistiskt säkerställda behandlingseffekter, både beträffande social ångest och livskvalitet. Resultaten förefaller också stå sig vid uppföljning ett år efter avslutad behandling.

Syftet med föreliggande studie är att jämföra den aktuella Internetbehandlingen med en redan etablerad behandling, kognitiv beteendeterapi i gruppformat. Så vitt vi vet har Internetbehandling för social fobi inte tidigare prövats inom klinisk psykiatrisk verksamhet. Ytterligare syften är därför att undersöka huruvida Internetbehandlingen är en effektiv behandlingsmetod för personer med social fobi som söker sig till primärvård eller psykiatrisk öppenvård, samt för ungdomar i åldern 16-18 år, som normalt inte behandlas inom vuxenpsykiatrin, men som i dagsläget inte erbjuds evidensbaserad behandling inom barnpsykiatrin.

Ett annat motiv bakom studien är att fördjupa kunskaperna kring Internet som behandlingsmedium inom psykiatrisk vård. Ett framtida eventuellt permanentande av en sådan resurs skulle markant öka tillgängligheten till effektiv behandling vid social fobi och utgöra ett tids- och kostnadseffektivt komplement till traditionell vård.

**2:2 Vilken primär vetenskaplig frågeställning ligger till grund för projektets utformning**

Om projektet kan karakteriseras som en hypotesprövning, ange den primära och eventuellt sekundära hypotesen. Hänvisning till mer detaljerad information för fackmän kan ske till bifogat protokoll eller forskningsplan enligt 2:1

2:1.

Studiens primära syfte är att, med öppen frågeställning, jämföra effekten av Internetbehandling (Internetadministrerad självhjälpsbehandling med terapeutstöd via e-post) med traditionell KBT-behandling i gruppformat.

**2:3 Redogör för resultat från relevanta djurförsök**

| För viss, främst medicinsk, forskning ange skälen till att djurförsök inte utförts. |
| --- |

Ej applicerbart.

**2:4 Redogör översiktligt för undersökningsprocedur, datainsamling och datas karaktär**

Av beskrivningen skall framgå hur studien planeras genomföras. Beskriv insamlade datas karaktär. Hur säkerställs datas tillförlitlighet (t.ex. kvalitetskontroll/monitorering )? - Vid enkäter och intervjuer skall beskrivas tillvägagångssätt och t.ex. frågors innehåll och hur slutsatser dras. Enkäter och skattningsskalor skall bifogas.

- För medicinsk forskning skall anges t.ex. typer av ingrepp, mätmetoder, antal besök, tidsåtgång vid varje försök, doser och administrationssätt för eventuella läkemedel och/eller isotoper, blodprovsmängd (även ackumulerad mängd vid multipla försök). Ange även om och på vilket sätt undersökningsprocedur m.m. skiljer sig från klinisk rutin. Ange proceduren för att ge den eventuella behandling efter studiens slut, som kan erfordras. Ange procedur för insamling av biologiskt material. Redogör för datakällor och procedurer vid behandling av personuppgifter. För mer detaljerad information kan hänvisning ske till bilagt protokoll eller forskningsplan enligt 2:1.

Strukturerad diagnostisk intervju genomförs av specialistläkare i psykiatri, eller av ST-läkare under handledning av specialistläkare i psykiatri. Rutinblodprov genomförs inkluderande blodvärde, sköldkörtelhormon, leverstatus och vätskebalans för att differentialdiagnostiskt kunna bedöma eventuell kroppslig sjukdom.

Studien kommer att ha 128 deltagare, där alla slumpas med likstor sannolikhet till antingen Internetbehandling eller gruppbehandling. De personer som slumpats till Internetbehandling väljer ett personligt användarnamn för inloggning till webbapplikationen som finns tillgänglig via webbplatsen www.psykiatrikarolinska.org. Applikationen innehåller administrering av självskattningsskalor, självhjälpsprogram, meddelandefunktion för kontakt med behandlare samt diskussionsgrupp med interaktionsmöjligheter med övriga deltagare.

Huvudkomponenten i Internetbehandlingen utgörs av ett strukturerat självhjälpsprogram i tio moduler. Programmet bygger på beprövade KBT-interventioner med följande innehåll: information om social fobi och kognitiv beteendeterapi; exponering (dvs att gradvis och under kontrollerade former utsätta sig för det som väcker obehag); kognitiv omstrukturering (dvs att lära sig identifiera, ifrågasätta och förändra dysfunktionella tankemönster); tekniker för att bryta handikappande självfokusering i sociala situationer samt återfallsprevention.

Deltagaren arbetar med självhjälpsprogrammet under 15 veckor och får stegvis tillgång till de olika modulerna. För att säkerställa att patienten tillgodogjort sig innehållet i programmet besvarar patienten, efter varje modul, ett antal frågor som sänds till behandlaren via webbplatsen. Under hela behandlingsperioden finns mölighet att kontakta behandlare för att få svar på eventuella frågor eller för att få stöd i tillgodogörandet av programmet.

De deltagare som slumpas till gruppbehandling kommer att erhålla totalt 15 veckovisa sessioner à 150 minuter. Varje grupp kommer att ha 6-7 deltagare och ledas av 1-2 behandlare och de centrala behandlingskomponenterna är desamma som i Internetbehandlingen. Innan gruppstart träffar patienten en behandlare för en individuell session. Därefter träffas gruppen för gemensamma sessioner till och med session 13. De sista två behandlingstillfällena är flexibla till utformningen och kan genomföras antingen individuellt eller i grupp. Tanken är att patienter och behandlare tillsammans avgör vilka behov som föreligger och vilket innehåll dessa avslutande sessioner bör ha.

Rekrytering av deltagare till studien kommer att ske konsekutivt. När tillräckligt många deltagare (se ovan) slumpats till gruppbetingelsen påbörjas behandlingen för denna grupp samt de deltagare som fram till dess slumpats till Internetbetingelsen. Därefter fortsätter rekryteringen av fler deltagare som på samma sätt slumpas till grupp- respektive Internetbehandling, fram till dess att minst 64 personer kommit att ingå i vardera betingelse.

Effektmått i studien utgörs av etablerade bedömar- och självskattningsskalor avseende social ångest, undvikande och nedstämdhet/depression. Bedömande läkare administrerar Global Assessment of Function (GAF), Clinical Global Impression (CGI) samt Work Questionnaire (WQ). Deltagaren fyller själv, via webbapplikationen, i följande självskattningsskalor: Liebowitz Social Anxiety Scale (LSAS-SR), Montgomery Åsberg Depression Rating Scale (MADRS-S), Social Phobia Scale (SPS), Social Interaction Anxiety Scale (SIAS), Quality of Life Inventory (QOLI) och Beck Anxiety Inventory (BAI). Uppföljande läkarbedömning sker vid behandlingens slut samt 6 månader efter avslutad behandling. Veckovisa mätningar under pågående behandling genomförs med MADRS-S och LSAS-SR. Mätningar med samtliga självskattningsskalor genomförs vecka 0, 15, och 6 månader efter avslutad behandling. Deltagarna erbjuds därefter möjlighet till kontinuerlig uppföljning med årliga självskattningar via Internet.

Primärt utfallsmått utgörs av genomsnittlig sänkning av LSAS-poäng vid mätning efter avslutad behandling samt vid uppföljning, i jämförelse med förmätning. Därutöver görs analys av gruppskillnader avseende enskilda skalor samt genomsnittlig effektstorlek på förändring över samtliga ingående skattningar.

**2:5 Redogör för om insamlat biologiskt material kommer att förvaras i en biobank**

*Med biobank avses biologiskt material från en eller flera människor som samlas och bevaras tills vidare eller för en bestämd tid och vars ursprung kan härledas till den eller de människor från vilka materialet härrör.*

| Redogör för var och hur prover som skall sparas förvaras, kodningsprocedurer och villkor för utlämnande av prover. Observera att i förekommande fall skall anmälan av biobank ske till Socialstyrelsen enligt lagen (2002:297) om biobanker i hälso- och sjukvården m.m. |
| --- |

Ej applicerbart.

**2:6 Redovisa tillgång till nödvändiga resurser under hela projektets genomförande**

| Ange vilka som har ansvaret (prefekt, verksamhetschef eller motsvarande) för forskningspersonernas säkerhet vid alla enheter/kliniker där patienter ingår samt att erforderliga ekonomiska och personella resurser finns tillgängliga. Intyg från dessa skall bifogas. |
| --- |

Verksamhetschef Peter Nordström.

**2:7 Journalföring, registrering och hantering av data**

Redogör för hur undersökningsprocedurer och eventuella ingrepp journalförs. Ange hur registrering och behandling av resultaten skall gå till. Om materialet skall kodas, ange proceduren, vem som förvarar kodlistor och vem eller vilka som har tillgång till dem, var de förvaras, hur länge samt om materialet kommer att anonymiseras eller förstöras. Används band- och videoinspelningar? Vilken tillgänglighet har datamaterialet? Hur förvaras det? Hur erhålls erforderligt sekretesskydd?

Data från formulär och skattningsskalor samt korrespondens mellan behandlare och patient kommer att registreras i webbapplikationen endast i relation till anonymt användarnamn och kan inte spåras till en enskild patient.

För alla patienter kommer sedvanlig journal att föras vid Psykiatri Centrum Karolinska.

Data från studien kommer att redovisas i avidentifierad form med resultat huvudsakligen på gruppnivå. Patientens identitet kommer alltså inte att användas i databearbetning eller sammanställning.

**2:8 Redogör för tidigare erfarenheter (egna och/eller andras)**

**av den använda proceduren, tekniken eller behandlingen**

Kognitiv beteendeterapi har i kontrollerade studier medfört goda resultat för en rad ångeststörningar och utgör idag förstahandsval vid behandling av social fobi. De behandlingskomponenter som i metaanalyser funnits mest betydelsefulla är exponering (dvs att gradvis och under kontrollerade former utsätta sig för det som väcker obehag eller ångest) och kognitiv omstrukturering (dvs att lära sig identifiera, ifrågasätta och förändra dysfunktionella tankemönster). Båda dessa komponenter kommer att inkluderas i föreliggande studie, bla genom övningar som deltagarna genomför på egen hand utifrån tydliga instruktioner i självhjälpsprogrammet respektive under gruppsessionerna.

Särskilt angeläget är att redovisning av risker för komplikationer görs tydliga och i förekommande fall med angivande av relevanta publikationer. Om ansökan avser fortsättning eller uppföljning av tidigare projekt, ange diarienummer samt datum för beslut av tidigare godkänd ansökan. Vid nya läkemedelsbehandlingar av patienter bör anges hur många patienter (med aktuell eller annan åkomma) som tidigare erhållit föreslagen eller högre dosering samt hur långa behandlingsperioder som studerats.

Att Internet kan betraktas som ett fungerande medium för psykologisk behandling har tidigare visats i studier som granskats och godkänts av Forskningsetiska kommittén vid Uppsala universitet (bland annat vid paniksyndrom: D.nr 02-247, depression: D.nr 99-498 samt tinnitus: D.nr 02-248). Tydliga effekter erhölls också i de tidigare nämnda, ännu opublicerade, behandlingsstudierna av social fobi (D.nr 02-555 och 03-636).

Som tidigare nämnts pågår vid Psykiatri Centrum Karolinska sedan ca ett år ett forskningsprojekt med studier av Internetbehandling för paniksyndrom. Dessa studier har granskats och godkänts av KI Forskningsetikkommittée Nord vid Karolinska sjukhuset och Regionala Etikprövningsnämnden i Stockholm (D.nr 03-042 resp 04-034/4). Se ovan för resultat. Den psykolog (Erik Hedman) som kommer att vara projektledare i föreliggande studie har tidigare erfarenheter av Internetbaserad självhjälpsbehandling.

#### 3. Uppgifter om forskningspersoner

**3:1 Hur görs urvalet av forskningspersoner**

*Med forskningsperson avses en levande människa som forskningen avser.*

Ange urvalskriterier (inklusion och exklusion). På vilket sätt kommer forskaren i kontakt med/får kännedom om lämpliga forskningspersoner? Ange om rekrytering sker från (egna, andras) tidigare eller pågående studier. Om annonsering sker, skall annonsmaterialet insändas som bilaga. Om t.ex. barn, eller personer som tillfälligt eller permanent inte är kapabla att ge ett eget informerat samtycke skall tillfrågas om deltagande i projektet, skall detta särskilt motiveras. Om vissa grupper (t.ex. kvinnor, barn eller äldre) utesluts från deltagande i projektet skall detta särskilt motiveras.

Studien är öppen för patienter från hela Stockholms län, såväl via självanmälan som med remiss från läkare vid exempelvis vårdcentral eller psykiatrisk öppenvårdsmottagning. Lägsta ålder för deltagande är 16 år. Information om studien kommer att sändas till andra vårdenheter samt, om ytterligare rekryteringsbehov föreligger, publiceras som annons (se bilaga 3) i dagspress. Efter intresseanmälan ges muntlig och skriftlig information om studien (se bilaga 4).

Deltagare ska uppfylla kriterier för social fobi enligt DSM-IV och kan deltaga i studien så länge de inte har annan betydande samsjuklighet. Exklusionskriterier utgörs av (a) annan dominerande Axel-I-diagnos (b) substansberoende/missbruk under de senaste 6 månaderna, (c) historia av psykossjukdom eller bipolär sjukdom, (d) egentlig depression enligt DSM-IV och samtidig totalpoäng på >20 på MADRS-S, (e) aktuell suicidrisk (>3 poäng på Item 9, MARDS-S), (f) primär eller allvarlig störning enligt Axel-II (dock ej fobisk personlighetsstörning), (g) påbörjad eller förändrad farmakologisk behandling mot ångest/depression under de senaste två månaderna, (h) annan pågående psykologisk behandling för social fobi, (i) tidigare kognitiv-/kognitiv beteendeterapi för social fobi. Om inga exklusionskriterier noterats görs läkarbedömning för eventuell inklusion i studien, följt av randomisering. Förhållandet mellan antalet remitterade respektive antalet självanmälda patienter förväntas till följd av randomiseringsprocessen att bli jämnt fördelat över de båda betingelserna. Vid utvärdering av resultaten analyseras även huruvida omständigheten att deltagaren blivit remitterad alternativt själv anmält sig påverkar behandlingsresultatet.

**3:2 Ange relationen mellan forskare/försöksledare och forskningspersonerna**

Behandlare (t.ex. läkare, psykolog, sjukgymnast) - forskningsperson (t.ex. patient, klient)

Kursgivare (lärare) - student

Arbetsgivare - anställd

Annan relation. Beskriv:

3:3 Redogör för det statistiska underlaget för studiepopulationens (ernas)/ undersökningsmaterialets(-ens) storlek

| Redovisa en statistisk styrka, så kallad ”power”-beräkning eller motsvarande överväganden för tydliggörande av studiens möjligheter att besvara frågeställningarna. |
| --- |

Vad gäller skillnaderna mellan de två betingelserna anses en effekt som är mindre än d=.50 inte vara av kliniskt intresse. Med 128 deltagare (64 per betingelse), och en alfa-nivå på 0.05, kan en effekt av denna storlek eller större statistiskt säkerställas med 80% power.

3:4 Kan forskningspersonerna komma att inkluderas i flera

studier samtidigt eller i nära anslutning till denna studie

| Ange om forskningspersonerna kan inkluderas samtidigt i flera studier eller i nära anslutning till denna studie. Ange i så fall projekttitel, forskningshuvudman, forskare som genomför studien (kontaktperson) samt diarienummer (om känt) för de övriga studierna. När avslutades ett eventuellt tidigare deltagande? |
| --- |

Nej.

3:5 Vilket försäkringsskydd finns för de forskningspersoner som deltar i projektet

| Det åligger forskningshuvudmannen att kontrollera att befintliga försäkringar täcker eventuella skador som kan uppkomma. |
| --- |

Eventuella skadeverkningar till följd av behandlingen omfattas av Patientförsäkringen.

**3:6 Vilken ekonomisk ersättning eller andra förmåner utgår till de forskningspersoner som deltar i projektet och när betalas ersättningen ut** (Utförligare beskrivning kan lämnas i bilaga)

Ersättning för obehag och besvär. Belopp (före skatt): 0 kr.

Ersättning för förlorad arbetsinkomst  Ja  Nej

Reseersättning  Ja  Nej

Befrielse från kostnader för läkemedel  Ja  Nej

Befrielse från andra kostnader. Vilka?

Andra förmåner. Vilka?

När betalas ersättningen ut?

Ingen ersättning betalas ut

### 4. Information och samtycke

4:1 Proceduren för och innehållet i den *information* som

lämnas då forskningspersoner tillfrågas om deltagande

| Beskriv hur och när information ges och vad den innehåller. Vem informerar? Normalt skall en kortfattad och lättförståelig skriftlig information ges. Denna skriftliga information skall bifogas ansökan. Om ingen eller ofullständig information ges, måste skälen för detta noggrant anges. *Forskningspersonen skall informeras om* - den övergripande planen för forskningen  - syftet med forskningen  - de metoder som kommer att användas  - de följder och risker som forskningen kan medföra  - vem som är forskningshuvudman och kontaktperson  - att deltagandet i forskningen är frivilligt och  - forskningspersonens rätt att när som helst avbryta sin medverkan. |
| --- |

Muntlig samt skriftlig patientinformation (se bilaga 4) ges vid bedömningssamtal hos psykiater/ST-läkare.

4:2 Hur och från vem inhämtas *samtycke*

Samtycke inhämtas vid bedömningssamtal hos psykiater/ST-läkare, där patienten skriver under patientinformationen. Vårdnadshavare till potentiella deltagare under 18 år är välkomna att delta vid bedömningssamtal om ungdomen själv så önskar. Skriftligt samtycke från vårdnadshavare anses dock inte nödvändigt för deltagande i studien (§18 Lag [2003:460] om etikprövning av forskning som avser människor).

Beskriv proceduren; vem som frågar, när detta sker och hur samtycket dokumenteras. Utförlig redovisning är särskilt viktig då barn eller personer med nedsatt beslutskompetens ingår i studien, likaså vid studier av en grupp (grupper), t.ex. föreningar, organisationer, företag, kyrkosamfund och församlingar eller arbetet i en skolklass.

### 5. Forskningsetiska överväganden

**5:1 Redogör för risker som deltagandet kan medföra samt möjliga komplikationer**

Detta kan vara t.ex. smärta, obehag eller integritetsintrång som projektet innebär eller kan innebära.

Har åtgärder vidtagits för att förebygga de risker som sägs ovan? Vilken beredskap finns att hantera dessa komplikationer? Ange metoder som kommer att användas för att efterforska, registrera och rapportera oönskade händelser.

Patienterna deltar frivillligt i undersökningen. Studien förutsätter att deltagarna är väl införstådda med att de kommer att slumpas till antingen grupp- eller Internetbehandling och att en eventuell förbättring kräver aktivt engagemang.

Följande etiska risker har identifierats:

1) Patienter som utöver social fobi även har annan samsjuklighet och inte bedöms lämpliga för Internetbehandling kan komma att intressera sig för självhjälpsprogrammet. Dessa kommer vid behov att erbjudas annan behandling vid Ångestprogrammet, Psykiatri Centrum Karolinska, eller hänvisas till annan psykiatrisk vård.

2) Med hänsyn till sekretess kommer all information att skyddas genom att deltagare i Internetbehandlingen tilldelas ett personligt användarnamn och lösenord. Detta användarnamn kan väljas fritt och därigenom är den data som finns i webbapplikationen endast kopplad till ett självvalt och anonymt användarnamn. På ett nyckeldokument förvarad på ett säkert sätt vid Psykiatri Centrum Karolinska finns patientens identitet kopplad till användarnamnet (se även punkt 2:7).

3) Studien inkluderar en för sammanhanget ny patientgrupp, nämligen ungdomar i åldern 16-18 år. Internetbehandling har så vitt vi vet inte tidigare prövats för denna patientgrupp vilket gör att det i dagsläget är svårt att uttala sig om i vilken utsträckning de kan komma att tillgodogöra sig behandlingen. Med veckovisa självskattningar av nedstämdhet och social ångest har vi dock en god möjlighet att följa behandlingsförloppet och om behov föreligger, vidta nödvändiga åtgärder.

5:2 Redogör för förutsebar nytta för de forskningspersoner som ingår i projektet

Studien medför vinster för patienterna såtillvida att de får ta del av psykologisk behandling (kognitiv beteendeterapi) som annars är svår att få pga bristande tillgång på utbildade KBT-behandlare.

5:3 Gör en egen värdering av förhållandet risk – nytta för de forskningspersoner som deltar

Riskerna i föreliggande studie bedöms som mindre än nyttan för de personer som deltar.

**5:4 Identifiera och precisera vilka etiska problem t.ex. risk – nytta**

**i ett vidare perspektiv som kan uppstå inom eller genom projektet**

Social fobi är ett ångestsyndrom som resulterar i social funktionsnedsättning och medför ett stort personligt lidande och en låg grad av livskvalitet för den drabbade. Individer med social fobi ligger under genomsnittet vad gäller utbildningsnivå och socioekonomisk standard, sannolikt som en konsekvens av sjukdomen. Det förekommer också en betydande samsjuklighet med övriga ångestsyndrom, depression och olika typer av missbruksproblematik. Om denna Internetbaserade självhjälpsbehandling visar sig användbar inom klinisk psykiatrisk vård kan tillgängligheten till effektiv behandling ökas och mänskligt lidande minskas. Med tanke på ovan nämnda allvarliga konsekvenser av ångestsyndromet skulle ett framtida permanentande av behandlingsformen också medföra stora vinster ur ett samhällsekonomiskt perspektiv.

### 6. Redovisning av resultaten

6:1 Hur garanteras forskningshuvudmannen och medverkande forskare

tillgång till data (anges vid t.ex. uppdragsforskning) och vem ansvarar för databearbetning och rapportskrivning

| Vid uppdragsforskning anges hur forskningshuvudmannen och medverkande forskare garanteras tillgång till data och vem som ansvarar för databearbetning och rapportskrivning. |
| --- |

Huvudsökande (forskningshuvudman) genomför och ansvarar för all databearbetning och rapportskrivning.

6:2 Hur kommer resultaten att göras offentligt tillgängliga

Kommer studien att insändas för publicering i tidskrift eller publiceras på annat sätt

| Ange i vilken form resultaten planeras offentliggöras samt tidsplan för detta. |
| --- |

Resultaten sammanställs och publiceras i vetenskaplig tidskrift.

6.3 På vilket sätt garanteras forskningspersonernas rätt till integritet när materialet offentliggörs/publiceras

| Beskriv procedurer eller metod för avidentifiering/anonymisering. Redovisas endast resultat på statistisk gruppnivå? |
| --- |

Resultat redovisas endast statistiskt på gruppnivå.

7. Redovisning av ekonomiska förhållanden och

beroendeförhållanden

7:1 Vid uppdragsforskning

| Ange uppdragsgivaren t.ex. vid klinisk läkemedelsprövning. |
| --- |

Namn:       Kontaktperson:

Adress:       Telefon/mobiltelefon:

| Ange uppdragsgivarens relation till forskningshuvudmannen/medverkande forskare, t.ex. anställningsförhållande. |
| --- |

**7:2 Redovisa eventuella ekonomiska överenskommelser med uppdragsgivare eller andra finansiärer (namn, belopp)**

Vid klinisk läkemedelsprövning kan hänvisning ske till ingånget avtal med sjukvårdshuvudmannen eller genom uppgift om föreslagen ersättning enligt överenskommelsen mellan Landstingsförbundet och LIF som bifogas. Separata överenskommelser med den/de som skall genomföra forskningen skall också redovisas. Om överenskommelserna inte är klara i sin helhet vid tidpunkten för ansökan skall belopp för studien/ersättning till kliniken/genomföraren och vad ersättningen skall täcka alternativt belopp per forskningsperson anges här. Vid studier där fler än en forskningshuvudman deltar skall principerna för och storleksordningen för ersättning för studien i sin helhet anges.

Ej applicerbart.

7:3 Redovisa forskningshuvudmannens och medverkande forskares intressen/tillgångar

| Redovisa de som kan tänkas påverka tilltron till objektiviteten i genomförande och rapportering (t.ex. aktieinnehav eller konsultuppdrag i finansierande företag). |
| --- |

Ej applicerbart.

### 8. Förteckning över bilagor

| Dokument som, i tillämpliga fall, skall bifogas om inte motsvarande information finns i blanketten har markerats med x. Markera de bilagor som skickas in med denna ansökan. | | | | |  |
| --- | --- | --- | --- | --- | --- |
| **Insänd med ansökan** | **Bil nr** | Beskrivning | **Klinisk läkemedels**  **prövning** | **Annan forskning** | |
|  | 1  p 1:5 | Deltagande forskningshuvudmän och medverkande forskare (kontaktpersoner) vid forskning där mer än en forskningshuvudman deltar | x | x | |
|  | 2  p 2:1 | För fackmän avsedd projekt/forskningsplan (protokoll), vid behov även för lekmän avsedd bilaga | x | x | |
|  | 3  p 3:1 | Annonsmaterial för rekrytering av forskningspersoner | x | x | |
|  | 4  p 4:1 | Skriftlig information till dem som tillfrågas | x | x | |
|  | 5  p 2:4 | Enkät, frågeformulär | x | x | |
|  | 6 | Gemensam EU-blankett (gäller fr.o.m. den 1 maj 2004), gäller även vid ändring | x |  | |
|  | 7 | Sammanfattning av protokollet på svenska | x |  | |
|  | 8 | Prövarhandbok alt. bipacksedel/produktresumé | x |  | |
|  | 9  p 2:6 | Intyg från verksamhetschef/motsv. om resurser | x | x | |
|  | 10 | CV för forskare (samma som p 1:3) med huvudansvar för genomförande (redovisa forskarens kompetens) | x | x | |
|  | 11  p 3:6 | Beskrivning av ersättning till forskningspersoner | x | x | |
|  | 12  p 7:1 p 7:2 | Överenskommelser med uppdragsgivare/finansiär om  t.ex. anställningsförhållanden, bidrag/ersättning till prövningsplats, sjukvårdshuvudman, forskningshuvudman eller forskare | x | x | |
|  | 13 | Tillstånd från strålskyddskommitté eller motsvarande | x | x | |

**9. Undertecknande**

| Behörig företrädare för sökande forskningshuvudman enligt p 1:2. |
| --- |

Ort:       Datum:

______________________________________________________________________________________________

Signatur

Namnförtydligande:

| Undertecknad forskare som genomför projektet (kontaktperson) enligt p 1:3 intygar härmed att forskningen kommer att genomföras i enlighet med ansökan. |
| --- |

Ort:       Datum:

______________________________________________________________________________________________

Signatur

Namnförtydligande:

**Anvisning för ansökan**

Denna ansökningsblankett används vid ansökan om etikprövning enligt lagen (2003:460)

om etikprövning av forskning som avser människor. Den är avsedd att användas för all

slags forskning där godkännande skall inhämtas från en etikprövningsnämnd. Blanketten

skall användas även vid begäran om rådgivande yttrande enligt 2 § förordning (2003:616)

med instruktion för regionala etikprövningsnämnder. Beroende på vilken forskning som

ansökan gäller kommer de uppgifter som efterfrågas nedan att ha olika relevans. Därmed

varierar också kravet på utförlighet i redovisningen av dessa. Markera på formuläret när uppgiften inte berör det aktuella projektet. Ansökan skall ifyllas så att den blir lättläst,

dvs. den skall inte vara handskriven och inte skriven med liten stil och kort radavstånd. Blanketten skall skrivas på svenska.

Observera att en ansökan aldrig är komplett (och därmed kan behandlas) förrän blanketten är korrekt ifylld och avgiften är betald.

***Ansökan med bilagor insändes i ett original och 16 kopior!***

#### Om denna blankett

Allt som skall fyllas i är markerat grått. Övrig förklarande text är låst och får inte ändras. De grå fälten expanderar allteftersom du skriver, det finns alltså obegränsat utrymme för din text. Vid utskrift syns enbart texten du fyllt i och ej de grå fälten som syns på skärmen, det är därför viktigt att kontrollera att allt verkligen är ifyllt. När du är klar skriver du ut blanketten, kompletterar med erforderliga underskrifter samt bilagor och skickar till den regionala etikprövningsnämnd till vars upptagningsområde forskningshuvudmannen hör, se www.forskningsetikprovning.se.
